# Ultrastructure damage of oviduct telocytes in rat model of acute salpingitis

**DOI:** 10.1111/jcmm.12548

**Published:** 2015-03-06

**Authors:** Jian Yang, Chi Chi, Zhen Liu, Gang Yang, Zong-Ji Shen, Xiao-Jun Yang

**Affiliations:** a Department of Obstetrics and Gynecology, The First Affiliated Hospital of Soochow UniversitySuzhou, China; b Lab Center, Medical College of Soochow UniversitySuzhou, China

**Keywords:** telocytes, acute salpingitis, tubal factor infertility, tubal ectopic pregnancy, inflammatory factors, stromal cells, rat model, oviduct, fibrosis, immunoregulation

## Abstract

Acute salpingitis (AS) is an inflammatory disease which causes severe damage to a subset of classically described cells lining in oviduct wall and contributes to interstitial fibrosis and fertility problems. Telocytes (TCs), a newly discovered peculiar type of stromal cells, have been identified in many organs, including oviduct, with proposed multiple potential bio-functions. However, with recent increasing reports regarding TCs alterations in disease-affected tissues, there is still lack of evidence about TCs involvement in AS-affected oviduct tissues and potential pathophysiological roles. We presently identified normal TCs by their characteristic ultrastructural features and immunophenotype. However, in AS-affected oviduct tissues, TCs displayed multiple ultrastructural damage both in cellular body and prolongations, with obvious loss of TCs and development of tissue fibrosis. Furthermore, TCs lose their interstitial 3-D network connected by homocellular or heterocellular junctions between TCs and adjacent cells. And especially, TCs connected to the activated immunocytes (mononuclear cells, eosinophils) and affected local immune state (repression or activation). Meanwhile, massive neutrophils infiltration and overproduced Inducible Nitric Oxide Synthase (iNOS), COX-2, suggested mechanism of inflammatory-induced TCs damage. Consequently, TCs damage might contribute to AS-induced structural and reproductive functional abnormalities of oviduct, probably *via*: (*i*) substances, energy and functional insufficiency, presumably, *e.g*. TC-specific genetic material profiles, ion channels, cytoskeletal elements, Tps dynamics, *etc*., (*ii*) impaired TCs-mediated multicellular signalling, such as homeostasis/angiogenesis, tissue repair/regeneration, neurotransmission, (*iii*) derangement of 3-D network and impaired mechanical support for TCs-mediated multicellular signals within the stromal compartment, consequently induced interstitial fibrosis, (*iv*) involvement in local inflammatory process/ immunoregulation and possibly immune-mediated early pregnancy failure.

## Introduction

Pelvic inflammatory disease (PID) is an inflammatory disease which frequently causes fertility problems among women of reproductive age 1. Among the pathogens, mycoplasma, Neisseria gonorrhoeae and Chlamydia trachomatis were prevail. In addition, organisms that are normal for vaginal flora were also considered as conditioned pathogen for PID. In clinic, acute PID causes tubo-ovarian abscess, pelvic peritonitis, endometritis and acute salpingitis (AS). In particular, AS often causes severe damage of a subset of oviduct cells, resulted in oedema and obstruction of the lumen, interstitial fibrosis and rigidity of the wall. Finally lead to decreased fertility capacity, such as tubal factor infertility (TFI) or tubal ectopic pregnancy (TEP) 1. Nevertheless, behind the known damage of classically described oviduct cells in AS 2, what else occurred simultaneously within the oviduct wall which contribute to structural and functional abnormalities is worthy of further investigation.

Telocytes (TCs) were recently identified as a peculiar type of stromal cells by Popescu *et al*. 3–12, in various mammal organs including the heart, lung, skeletal muscle, urinary tract, uterine and fallopian tubes, *etc*. (www.telocytes.com) 3–27. Telocytes have particular ultrastructure which can be clearly distinguished from interstitial cell of Cajal (ICC) and other type of interstitial cells 15. Morphologically, TCs have a small cellular body with characteristic prolongations named telopodes (Tps), which composed of thin segments (podomers) and dilated segments (podoms) 8. Several roles have been proposed for TCs, including intercellular signalling, spreading slow waves generated by the pacemaker ICC, stem cells (SCs) mediated tissue reparation, tissue homostasis/ angiogenesis and immunoregulation 28. Previous studies on TCs mainly focused on the identification, distribution, ultrastructure and immunophenotype in various normal tissues or organs. Recently, TCs alterations in disease-affected tissues were reported in fibrotic lesions of skin, cardiac, ulcerative colitis, Crohn’s disease and gallstone disease 29–34. Furthermore, we have demonstrated that in endometriosis-affected oviduct tissues, ultrastructural damage and significant loss of TCs might contribute to interstitial fibrosis and subsequent oviduct dysfunction 35. Nevertheless, whether AS also caused TCs damage and led to structural and functional abnormalities of oviduct, further engaged in poor reproductive outcome, need more detailed evidence.

On the basis of our previous observations 35, the current hypothesis is that, in exposure to acute inflammatory, oviduct TCs underwent cell damage and cause structural alterations, further induce functional abnormalities of oviduct. In support to this hypothesis, we conduct a comparative study on ultrastructural damage of TCs in SD rat model of AS-affected and -unaffected oviduct tissues, respectively, together with determination of inflammatory state. It is hoped that the results could add more information on AS-associated TFI and TEP.

## Materials and methods

### Animals

Three-month-old virgin, female, Sprague–Dawley rats (200–250 g) were provided by the Laboratory Animal Center of Soochow University (animal certification numbers: 0102261). All rats were maintained in specific pathogen free conditions under environmentally controlled temperature of 21 ± 1°C, and were acclimatized to 12 hrs day/night cycle for at least 10 days before being used for experiments. The study protocol was approved by the Institutional Ethics Review Board of Soochow University.

### Microorganism culture

*Escherichia coli* (CMCC 44102; Fu Xiang biotechnology, Shanghai, China) was used for induction of AS. Single colony of a pure culture of *E. coli* was transferred and incubated overnight for 18 hrs at 37°C, aerobically in the dark. For stock cultures, beef extract decoction agar slants were inoculated and similarly incubated, until an optical density equivalent to 10^9^ cfu/ml were acquired 36.

### Animal surgery

Thirty rats were randomly divided equally into AS and sham control group. Rat model of AS-affected oviduct was established 2. Briefly, all rats received anesthetization with sodium pentobarbital intraperitoneally (50 mg/kg; Fuyang Pharmaceutical Factory, Fuyang, China) and the surgery was performed under aseptic conditions. After entering the abdominal cavity *via* lower midline abdominal incision, both oviducts were identified and bacteria liquid (0.1 ml, 2 × 10^7^ *E. coli*) were injected into its lumens in AS group. Applying the same amount of sterile saline instead of bacterial liquid served as the sham controls. Then, the abdominal wall was closed and maintained in the same conditions.

### Samples gathering

At the seventh day after surgery, when rat model of AS were successfully developed 2, all rats from both groups were killed and both sides of oviducts tissues were obtained. Freshly dissected tissue segments (1 cm^3^) were fixed in 10% formalin and embedded in paraffin. Serial sections (5 μm) were subjected to haematoxylin and eosin, immunohistochemistry (IHC) and immuofluorescence experiments. The rest of fresh oviduct tissues (1 mm^3^) were used for ultrastructural observation of TCs by transmission electron microscope (TEM).

### Measurement of neutrophils infiltration

To evaluate neutrophils infiltration, sections were processed for routine IHC using rabbit anti-rat polyclonal CD177 (1:100; cat. no. bs-1482R; Bioss, Beijing, China). Omission of the primary antibody served as the negative control.

### Quantitative determination of inflammatory factors

iNOS and COX-2 were quantitatively measured by single-labelled immunofluorescence. Briefly, sections were incubated with primary anti-rat antibodies: rabbit polyclonal anti-iNOS (1:100; cat no.sc-649); mouse polyclonal anti-COX-2 (1:100; cat no.sc-166475; all from Santa Cruz Biotechnology, Santa Cruz, CA, USA). Then FITC-goat anti-rabbit/mouse IgG (1:100; cat. no. BA1105/BA1101; all from Boster, Wuhan, China) was added. Finally, the sections were mounted with antifade medium (1:1000; cat no. p0126; Beyotime, Shanghai, China). Immunofluorescence intensity was quantitatively measured by laser confocal scanning microscope (TCS-SP2; Leica Lasertechnik, Heidelberg, Germany). Images were converted to greyscale and pixel values of positive-stained areas were used for staining intensity. Total staining intensity per selected area was calculated by multiplying the number of pixels/area with the area mean intensity. At least 500 cells obtained from three separate areas/microscopical fields for iNOS (COX-2, respectively) were analysed for each experimental condition using Image-Pro plus software (version 6.2, Media Cybernetics, Inc, Bethesda, MD, USA) 37.

### TCs immunodiagnostics

Generally, double-labelled immunofluorescence using CD34 in combination with PDGFR alpha or beta, c-kit and vimentin was the available choice for TCs immunodiagnostics 15,29. Representative serial sections were processed for the primary antibodies in pairs (vimentin *versus* CD34, vimentin *versus* c-kit): mouse anti-rat monoclonal vimentin (1:100; cat no. BM0135), rabbit anti-rat polyclonal CD34 (1:100; cat no. BA0532), rabbit anti-rat polyclonal c-kit (1:100; cat no. BA0467-1). Then FITC-goat antimouse IgG for vimentin (1:50; cat no. BA1101), CY3-goat anti-rabbit IgG for CD34 (1:50; cat no. BA1032), TRITC-goat anti-rabbit IgG for c-kit (1:50; cat no. BA1090) were added (all from Boster). Finally, counterstained with DAPI (1:50; cat no. C1002) and mounted with antifade medium (1:1000; cat no. p0126; both from Beyotime). Sections were observed under fluorescence microscope (Olympus BX51; Olympus, Tokyo, Japan). Telocytes was the only kind of cell which were double-positive for vimentin and CD34, with typical TCs morphology and intact nuclei 35. The number of TCs was identified in 10 randomly selected microscopic high-power fields per section (40 × 10 original magnification), and statistically analysed in 30 AS-affected and -unaffected sections respectively 29,32.

### Ultrastructure observation

Tissue fragments (1 mm^3^) were processed for routine epon-embedding procedures 38. The selected regions were cut into ultra-thin sections (60 nm) and then received inspection under TEM (Hitachi H-600; Hitachi, Tokyo, Japan), with necessary adjustments of representative microphotos by Adobe Photoshop (version 7; Adobe Systems, Inc., San Jose, CA, USA).

### Statistical analysis

Values of the number of TCs, immunofluorescence intensity of iNOS and COX-2 were presented as mean ± SD, and analysed with Student’s *t*-test by SPSS (version 13; SPSS Inc., Chicago, IL, USA). *P* < 0.05 was considered to be statistically significant.

## Results

### Histology and CD177 IHC observation

**Figure 1:**
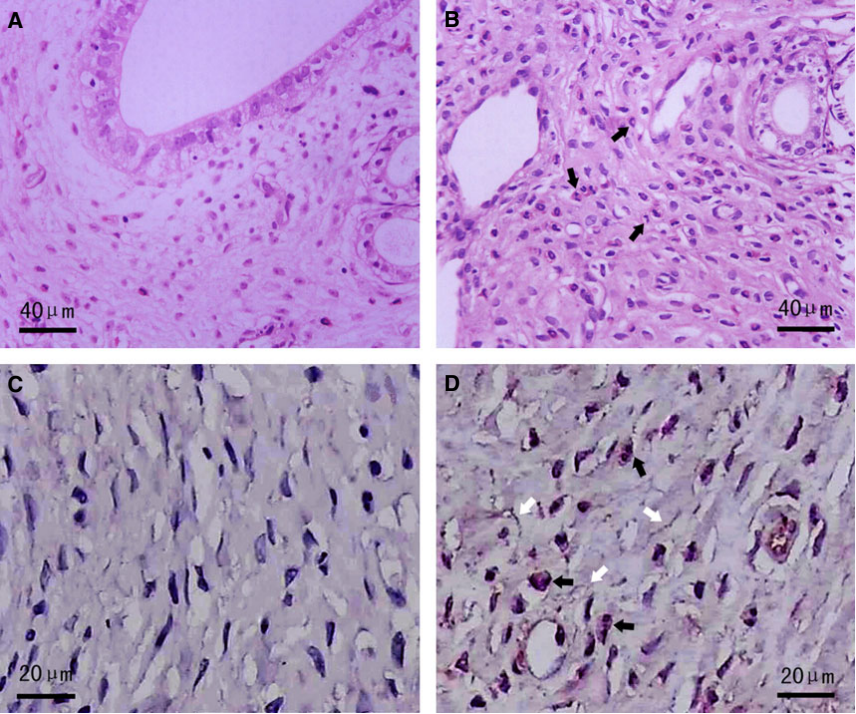
Haematoxylin and eosin and CD177 IHC staining in AS-affected and -unaffected oviduct tissues. (A) Normal oviduct tissues from the sham group displayed no obvious changes (haematoxylin and eosin staining). (B) Representative microphotographs of acute inflammation in AS-affected oviduct tissues, including SMCs and capillaries swelling, inflammatory congestion and exudation, interstitial oedema and infiltration of neutrophils (black arrows) (haematoxylin and eosin staining). (C) Totally negative CD177 immunostaining indicating no obvious infiltration of inflammatory cells in sham group. (D) Extensive infiltration of neutrophils (black arrows) as indicated by strong positive CD177 immunostaining, together with interstitial fibrosis (white arrows) in AS-affected oviduct tissues.

At the seventh day after surgery, on gross examination, 30 oviducts (15 rats) in AS group demonstrated dilated lumen, wall congestion and peritubal adhesions. On microscopic examination, specimens from AS group presented with acute inflammation reaction as follows (*i*) smooth muscle cells (SMCs) and capillaries swelling, inflammatory congestion and exudation, interstitial oedema and infiltration of neutrophils (Fig.1B); (*ii*) extensive infiltration of numerous neutrophils (Fig.1B and D), as indicated by strong positive CD177 immunostaining, together with interstitial fibrosis, mainly within the mucosal and muscular layers of oviduct (Fig.1D). However, in sham group (30 oviducts/15 rats), nearly normal histology was observed (Fig.1A), with no evidence of neutrophils infiltration proved by totally negative CD177 IHC (Fig.1C).

### Quantification of inflammatory factors

**Figure 2:**
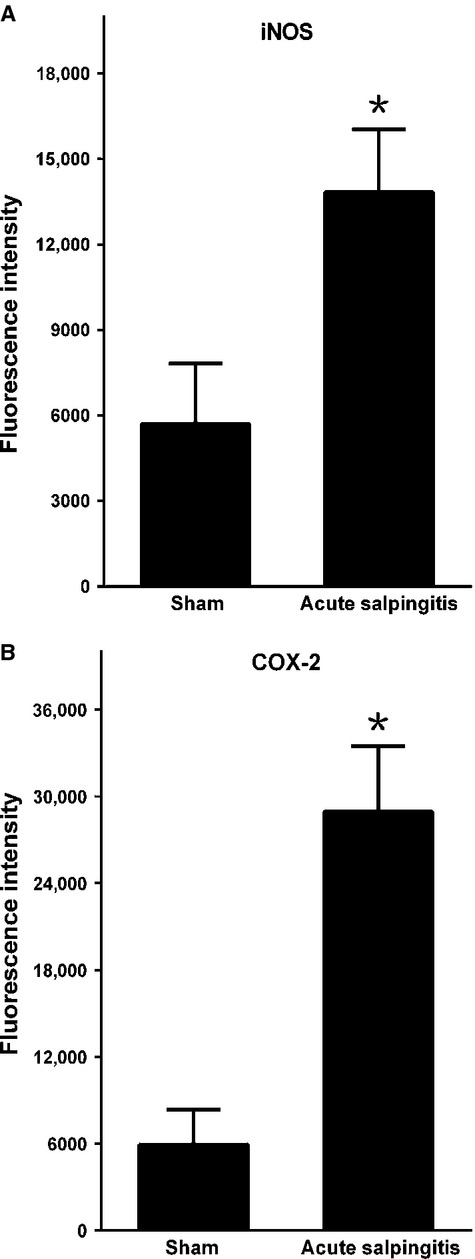
Inflammatory factors in AS-affected oviduct tissues were significantly higher than that in sham control. (A) iNOS, (B) COX-2. *P < 0.05 *versus* sham control; error bars = SD.

By using single-labelled immunofluorescence, the expression of iNOS and COX-2 were visualized. Quantitative analyse under laser confocal scanning microscope showed that in AS group, the expression level of iNOS and COX-2 were significantly higher than that in the sham group: iNOS (*P* = 0.000; Fig.2A) and COX-2 (*P* = 0.000; Fig.2B), thus confirmed overproduced inflammatory substance in AS-affected oviduct tissues.

### TCs distribution

**Figure 3:**
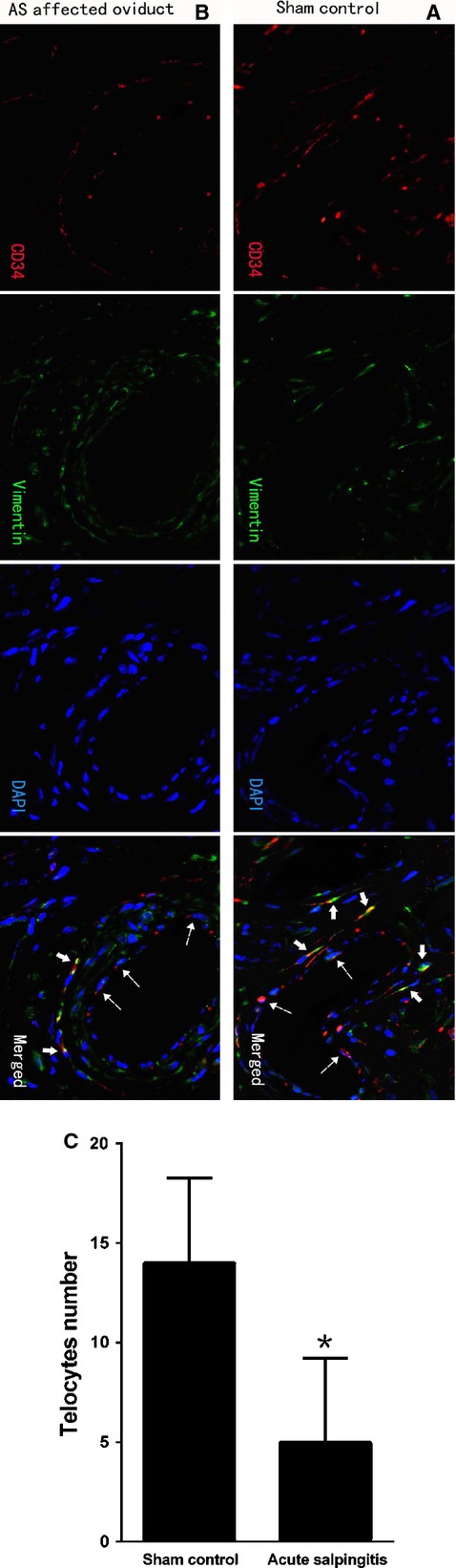
TCs immunodiagnostics by double-labelled immunofluorescence. Dotted arrows indicated CD34-positive vascular endothelial cells. Negative c-kit staining was not shown here; scale bar = 20 μm. (A) CD34 (red) in moniliform cells overlying vimentin (green) cells with DAPI counterstaining (blue) in sham control (solid arrows), indicated the existence of perivascular TCs with special immunophenotype of CD34/vimentin double-positive. (B and C) CD34/vimentin double-positive cells with specific TCs morphology and well-defined nuclei was significantly less densely stained, reduced, sparse or completely absent (solid arrows) in AS-affected oviduct tissues. A statistically significant decrease in the mean number of TCs occurred (*P* = 0.000). *P < 0.05 *versus* sham control; error bars = SD.

In the sham group, by using double-labelled immunofluorescence, rich amount of CD34 positive cells (red) were detected, overlapped with vimentin positive immunofluorescence (green) in the merged images. Meanwhile, there were no cells demonstrated co-expression of vimentin and c-kit (images not shown). Moreover, CD34/vimentin double-positive cells demonstrated a small cell body (DAPI, blue), with two or more extremely long/thin prolongations surrounding the capillaries, as well as double immunofluorescence in its full length (Fig.3A), thus confirmed the existence of TCs. However, in contrast, in sections from AS-affected oviduct tissues, CD34/vimentin double-positive cells with well-defined DAPI nuclei, were obviously less densely stained, reduced, sparse or completely absent (Fig.3B). A statistically significant decrease in the mean number of TCs occurred when compared with the sham control (*P* = 0.000), which demonstrated rich double immunostaining for CD34/vimentin (Fig.3C).

### Ultrastructure observation

**Figure 4:**
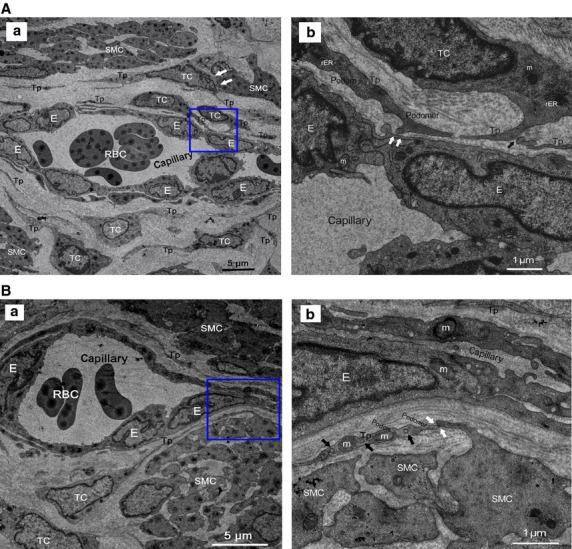
Normal TCs and Tps distributed in perivascular space or SMCs bundles. RBC: red blood cells; E: vascular endothelial cells. (A) Perivascular TCs. (a) A number of TCs, by their extremely long/thin Tps, surrounded and formed an almost complete circle around capillaries. Tps was composed of podoms and podomers. TCs established heterocellular contact with SMCs (white arrows). Mitochondria (m), rough endoplasmic reticulum (rER) and secretory granules can be observed. (b) Higher magnification of the boxed area; TCs frequency formed homocellular junctions (black arrow) and heterocellular junctions (white arrows) with E. (B) Perivascular TCs. (a) TCs surrounded capillaries and scattered among SMCs. b higher magnification of the boxed area. (b) Abundant mitochondria (m) and microvesicles (black arrows) contained in podom. Tps established heterocellular contacts with SMCs (white arrows).

**Figure 5:**
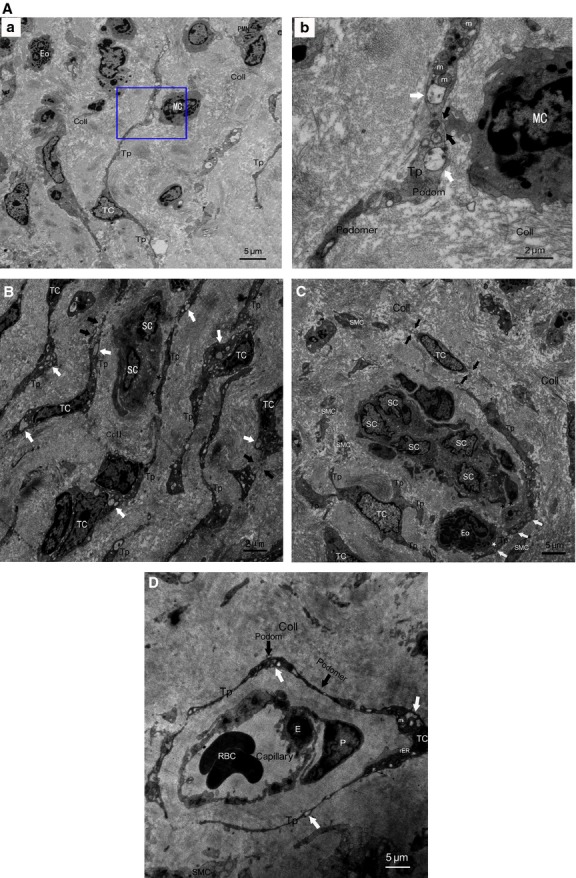
TCs and Tps damage in AS-affected oviduct tissues, accompanied by excessive amount of collagen fibres (Coll) and tissue fibrosis. (A) Intercellular connection between damaged TCs and activated mononuclear cells (MC). (a) Degenerated Tps established closed contact to activated MC which contained dense secretory granules, together with granulocyte infiltration, mainly eosinophils (Eo) and neutrophils (PMN). b higher magnification of the boxed area; (b) synapse (black arrows) between activated MC and degenerated Tps, which contained lots of swollen mitochondria (m) and vacuoles (white arrows), thus indicating degeneration, functional insufficiency of TCs and involvement of TCs in local immunoregulation. (B) Degeneration, discontinue or dissolution of TCs and Tps (black arrows), with cytoplasmic vacuolization (white arrows), accompanied by nearly normal scattered putative stem cells (SCs). Intercellular contacts between TCs and SCs was getting wider or disappeared (black asterisks). (C) Disrupted TC-SC niches which composed of a group of damaged Tps and putative SCs in myosalpinx, with heterocellular contacts getting wider or disappeared between Tps and SCs (black asterisks), Tps and activated Eosinophils (Eo) (white asterisk) with dense secretory granules respectively. Degeneration, discontinue or dissolution of TCs and Tps (black arrows), with swollen mitochondria (m), cytoplasmic vacuolization (white arrows) in Tps, swollen and dissolution of SMCs can be observed. (D) Severely damaged perivascular TCs and Tps, with swollen mitochondria (m), rough endoplasmic reticulum (rER) dilatation and cytoplasmic vacuolization (white arrows), together with damaged endothelial cell (E) and pericytes (P).

The existence of a novel and peculiar interstitial cell type named TCs in oviduct tissues was observed under TEM. Telocytes had a slender piriform/spindle/triangular-shaped cellular body, with extremely long/thin and moniliform Tps, frequently two or more per TCs (Figs4A and 5A–D). Moreover, Tps showed specific features of uneven calibre, alternated with thin segments (podomers) and dilated segments (podoms) (Figs4A, B and 5A, D). In addition, the cellular body and podoms were rich in mitochondria, rough endoplasmic reticulum, caveolae and cytoskeletal elements (Figs4A and 5A and D).

Telocytes were observed frequently enwrapping whole capillaries with their long Tps, or scattered in SMCs bundles in mucosa and muscular layer of oviduct (Figs4A, B and 5D). And by their Tps, TCs developed complex homocellular (between TCs) and heterocellular junctions with nearby cells: capillaries (Fig.4A), SMCs bundles (Fig.4B) and activated immunocytes with dense secretory granules (mononuclear cells, MC; eosinophils, Eo; Fig.5A and C). On the other hand, TCs organized a unique 3-D network by interconnecting different cell types and potentially affecting any of them with its specific Tps, thus occupied strategic positions and provided supporting materials or structural basis in the stromal compartment.

In AS-affected oviduct tissues, TCs was obviously decreased or lost, severely damaged/degenerated with multiple ultrastructural abnormalities, such as loss of organelles, numerous swollen nucleus, mitochondria and rough endoplasmic reticulum dilatation, cytoplasmic vacuolization, discontinue or dissolution of Tps, swollen or loss of intercellular junctions (Fig.5A–D). The original slender cellular body and Tps sometimes become enlarged owing to presence of a lot of vacuoles inside (Fig.5A–D). In addition, TCs was also found in close relationship with nearly normal scattered (Fig.5B) and damaged putative SCs (Fig.5C) within myosalpinx. Disrupted TC-SC niches (TC-SCNs) which composed of a group of damaged Tps and putative SCs can be observed. Heterocellular contacts become wider or disappeared between Tps and SCs, Tps and activated immunocyte (Eo) with dense secretory granules respectively (Fig.5C). However, the relation between TCs and SCs can be clearly observed in our previous result, in which, ‘Fig. 6E’ demonstrated that the damaged TCs surrounded a cluster of putative normal SCs by their TPs, make intact TC-SCNs, with heterocellular contacts between Tps and SCs 35. Meanwhile, excessive collagen fibres confirmed formation of interstitial fibrosis (Fig.5A–D).

## Discussion

Acute salpingitis was the most important component of PID spectrum 1, and it can cause severe damage to a subset of cells lining in the oviduct wall, mainly including the ciliated cells and classically described interstitial cells in the stromal compartment, such as fibroblasts, myofibroblasts, mast cells, macrophages, immune cells and ICC, each of them contributes essential role and engaged in the whole reproductive process. Nevertheless, as a new type of interstitial cell, pathophysiological role of TCs in AS-induced tubal factor fertility problems remains unknown. Herein, the successfully developed rat model of AS displayed acute inflammation, infiltration of numerous neutrophils and interstitial fibrosis, together with overproduced iNOS, COX-2 in oviduct tissues, suggested mechanism of inflammatory-induced TCs damage.

To date, a number of studies on TCs have yielded constant results regarding the identification, distribution, ultrastructure and immunophenotype in various normal cavitary and non-cavitary mammal tissues and organs, including heart 4,39, lungs 8,13,27,40, skeletal muscle 9, skin 12,14, gut 15, mammary glands 16, urinary tract 17,18, genital tract 19–21 and fallopian tubes 19,22–24. Telocytes are completely different from other related cells, *e.g*. fibroblasts, mesenchymal SCs, lymphocytes or endothelial cells, with specific roles in cell signalling, tissue or fibrosis remodelling, angiogenesis, physical and/or chemical sensors, not only by their specific ultrastructural configuration 3,8, TC-dominated genome, proteomic profiles and microRNA signature 41–45, immunophenotype 46, but also from different dynamics of Tps in terms of adherence, spreading/extension and ramification 47,48. Furthermore, in human atrial and ventricular, functionally competent K^+^ channels are present in TCs and might have significant implications in myocardial physiopathology 49. In uterine tissues, TCs was reported to have T-type calcium channels and participate in generation of endogenous bioelectric signals to regulate contractile of SMCs during pregnancy and labour 50,51.

Presently, normal oviduct TCs was identified and confirmed the literatures description 19,22–24, with TCs being located mainly in mucosa and muscular layer of oviduct tissues. Telocytes can be clearly distinguished from other type of interstitial cells, based on their distinct ultrastructure and immunophenotype (CD34-positive/vimentin-positive/c-kit-negative). Furthermore, by homocellular and/or heterocellular (planar or point) junctions between its specific Tps and nearby cells, *e.g*. capillaries, SMCs, activated immunocytes and SCs, TCs formed a complex 3-D extracellular network within the stromal compartment. Meanwhile, TCs occupied a critical position in such specific 3-D structure, indicated roles of mechanical support for intercellular signalling, possibly through chemical (short distance) interaction and physical (long distance) interaction 25. In detail, TCs frequently surround whole capillaries with its long/thin Tps, thus suggested potential participation in local homostasis and angiogenesis. Furthermore, their distribution among SMCs bundles suggested potential involvement in neurotransmission regarding muscle contraction, connection with activated MC/Eo suggested potential roles in local immunoregulation, participation in construction of TC-SCNs suggested potential roles in SCs mediated tissue repair/regeneration, enwrapped in collagen bundles suggested a potential roles in the process of fibrotic remodelling.

Currently, following our recent study on TCs in endometriosis-affected oviduct tissue, we herein reported for the first time that TCs displayed inflammatory-induced extensive ultrastructure damage, with obvious cell loss, decrease or almost complete absence in AS-affected oviduct tissues. Unfortunately, owing to degeneration, swollen or enlarged, discontinue or dissolution of TCs and Tps, we could not perform comparative morphometric analysis between AS-affected and -unaffected oviduct tissues, *e.g*. Tps length, podomer thickness and podoms’ diameters under TEM, as previously reported in human normal myometrium 50. Interestingly, increasing reports now focus on TCs alterations in disease-affected tissues, such as in fibrotic lesions of skin, cardiac, ulcerative colitis, Crohn’s disease, gallstone disease and endometriosis-affected oviduct tissues 29–35. These studies provided evidence for involvement of TCs in the fibrotic process of multiple organs. Nevertheless, pathological roles of TCs in disease-affected tissues still remain to be fully understood, although multiple bio-functions has been proposed for normal TCs 5,8–10,12,26,29,31,40,52,53. Accordingly, TCs loss might have important pathophysiological implications in AS-induced structural and reproductive functional abnormalities of oviduct, probably *via* the follows, although none of them has yet been proven definitively.

First, ultrastructure damage or loss of multiple subcellular organelles within cellular body and Tps, will inevitably influenced metabolic pathways, including substances synthesis and energy metabolism, presumably, *e.g*. TC-specific genome, proteomic profiles and microRNA signature, ion channels, neurotransmitter, cytoskeletal elements, *etc*. Then substances and energy insufficiency will decrease basic activities of TCs, such as electrophysiology, dynamics of Tps in terms of adherence, spreading/extension and ramification. Second, swollen or loss of intercellular contacts between TCs and neighbouring cells (capillaries, SMCs, activated immunocytes, SCs, *etc*.), will impair multicellular signalling and proposed bio-functions, regarding homeostasis/angiogenesis, tissue repair/regeneration, neurotransmission/muscular contraction, immunoregulation.

Third, TCs and Tps damage will change its ultrastructural configuration and the strategic extracellular 3-D architecture, which was structural basis or mechanical support for the guidance of cell migration (SCs, activated immunocytes, *etc*.), for multicellular signals integrating and coordination, and for the correct organization of extracellular matrix components. Derangement of 3-D network will alter the spatial relationships of Tps with neighbouring cells; induce disintegrating of different structural components of interstitium, until finally cause interstitial fibrosis. Interestingly, it was reported that, TCs loss might precede the onset of fibrotic remodelling of the intestinal wall, rather than being merely a consequence of the fibrotic process 33. Nevertheless, now it is still difficult to tell whether TCs loss happened earlier than the onset of oviduct fibrosis.

Lastly, as we know, a special immune circumstance within oviduct was essential to a successful fertilization, development, transportation until a positive pregnancy outcome. However, abnormal local immune state which were structurally contributed and regulated by intercellular contact between impaired TCs and activated MC/Eo, might functionally affect (repression or activation) release of various cytotoxic cytokines and enzymes into intratubal fluids, which will potentially lead to sperm phagocytosis, impaired fertilization, defective early embryo growth, abnormal transportation and implantation, finally cause immune-mediated failure of earlier pregnancy, including TFI and TEP.

Even more importantly, the findings of inflammation-induced TCs damage in infection based inflammatory AS was consistent with, and further strengthen the idea of a broad involvement of TCs in our previously reported endometriosis-affected oviduct tissue, which was an aseptic inflammatory, ischaemic, hyperoestrogenic condition 54. In which, extensive ultrastructural damage (degeneration, discontinue, dissolution and destruction), significant decrease or loss of TCs and interstitial fibrosis were observed, together with elevated level of iNOS, COX-2, lipid peroxides and oestradiol, thus suggestive of inflammation and ischaemia-induced TCs damage, and potential toxic effect of hyperoestrogenic state on oviduct TCs which express oestrogen/progesterone receptors 23. Nevertheless, TCs damage in both inflammatory disease-affected tissues was considered to contribute to structural and reproductive functional abnormalities of oviduct, similarly *via* above-mentioned mechanisms. In addition, similar to the role of local immunoregulation through heterocellular junctions between TCs the activated immunocytes (MC, Eo) in AS, synapse between Tps and activated mast cells, also indicated TCs involvement in local inflammatory response and possibly associated tubal factor fertility problems in endometriosis-affected oviduct tissues 35.

In conclusion, this is the first report on inflammatory-induced ultrastructural alterations of TCs in AS-affected oviduct tissues, presented with obvious damage and loss of TCs and its intercellular junctions accompanying the fibrotic remodelling of oviduct wall. And especially, TCs was found to participate in local immunoregulation. On the basis of these observations, TCs damage might further cause: (*i*) substance and energy insufficiency with deactivation of basic cell bio-functions, *e.g*. TC-specific genetic material profiles, ion channels, cytoskeletal elements, Tps dynamics, *etc*., (*ii*) impaired TCs-mediated multicellular signalling, such as homeostasis/angiogenesis, tissue repair/regeneration, neurotransmission, (*iii*) derangement of 3-D network and impaired mechanical support for TCs-mediated multicellular signals within the stromal compartment, consequently induced interstitial fibrosis, (*iv*) involvement in local inflammatory process/ immunoregulation and possibly immune-mediated early pregnancy failure. Nevertheless, it will be of great importance to investigate the underlying molecular and cellular mechanisms targeting oviduct TCs, and TCs-mediated pathological alterations, such as differentiation/activation of fibroblasts and immunocytes. Meanwhile, further functional studies are necessary to better identify the pathophysiological role of TCs in the setting of different oviduct disease.
